# Radiotherapy for postoperative groin lymphatic fistulas: A systematic review of clinical outcomes and treatment planning

**DOI:** 10.1016/j.ctro.2026.101274

**Published:** 2026-08-29

**Authors:** Nicolas Vial, Salvatore Cozzi, Daniele Brenna, Giulio Brignoli, Marialuisa Puglisi, Franscesco Mosè Castronovo, Davide Giovanni Bosetti, Letizia Deantonio, Francesco Martucci, Calin-Simion Toma-Micle, Myriam-Ines Gana, Bertrand Le Roy, Celine Chauleur, Paola Canino, Thomas Zilli

**Affiliations:** a Radiation Oncology, Oncology Institute of Southern Switzerland (IOSI), EOC, Bellinzona, Switzerland; b Department of Radiotherapy, University Hospital of Saint-Etienne, Saint-Priest-en-Jarez, France; c Faculty of Medicine, Jean Monnet University, Saint-Priest-en-Jarez, France; d Medical Oncology, Oncology Institute of Southern Switzerland (IOSI), EOC, Bellinzona, Switzerland; e Facoltà Scienze Biomediche, Università della Svizzera Italiana (USI), Lugano, Switzerland; f Department of Digestive and Oncological Surgery, University Hospital of Saint-Etienne, Saint-Priest-en-Jarez, France; g Department of Gynecology and Obstetrics, University Hospital of Saint-Etienne, Saint-Priest-en-Jarez, France; h Faculty of Medicine, Geneva University, Geneva, Switzerland

**Keywords:** Lymphatic fistula, groin, postoperative wound complication, radiotherapy

## Abstract

Postoperative groin lymphatic fistulas are a common complication of vascular surgery and radical lymph node dissection, which may occur in more than 10% of patients. While conservative management remains the standard first-line approach, refractory cases often require invasive interventions. Low-dose radiotherapy has emerged as a potential alternative, despite limited clinical adoption and an incompletely understood mechanism of action. This systematic review evaluates the available evidence on radiotherapy for postoperative groin lymphatic fistulas, focusing on clinical outcomes, safety, and treatment characteristics.

A systematic review was conducted in accordance with PRISMA guidelines, searching MEDLINE via PubMed from inception to June 2026. Studies reporting radiotherapy use for postoperative groin lymphatic fistulas in human subjects were eligible. Extracted data included study and patient characteristics, radiotherapy parameters, and clinical outcomes.

Twelve retrospective studies published between 1978 and 2026 were included, encompassing 448 patients. Radiotherapy schedules varied in timing (7.5–29 days post-surgery), dose per fraction (0.06–3 Gy), and total prescribed dose (0.5–20 Gy). Most protocols targeted the lymphocele and adjacent tissues, with a median treatment volume of approximately 585 cc. Fistula closure rates ranged from 67% to 100%, with complete response achieved within 5–27 days. Side effects were limited; one study reported a 2.4% incidence of secondary pelvic malignancies potentially attributable to radiotherapy.

Radiotherapy appears effective for postoperative groin lymphatic fistulas, achieving high closure rates with low-dose, short-course regimens. However, substantial heterogeneity and the absence of prospective evidence underscore the need for standardized protocols and future prospective studies.

## Highlights

• This review synthesizes evidence on radiotherapy for groin lymphatic fistulas.
• Twelve retrospective studies (448 patients) show closure rates of 67–100%.
• Low-dose regimens (≤0.5 Gy/fraction) match efficacy of higher-dose protocols.
• Treatment protocols vary widely, with no standardized dose or target volume.
• Prospective trials are needed to define optimal dose, timing, and volumes.

## 1 Introduction

Lymphatic fistulas are a frequent complication after vascular surgery and radical lymphadenectomy, especially in the groin [1], [2], [3], [4], with reported incidences ranging from 10% to 15% following vascular procedures [5], [6], [7]. These fistulas arise from lymphatic leakage and may lead to significant clinical consequences, notably local wound complications and systemic infections, which represent considerable challenges in postoperative management. Currently, there is no universally accepted definition of lymphatic fistula [8]. Although no consensus definition exists, lymphatic fistulas are commonly defined as drainage exceeding 50 mL per 24 h persisting beyond five postoperative days [9], [10]. The optimal management strategy for lymphatic fistulas refractory to conservative treatment, including negative pressure wound therapy [11], remains controversial. Available invasive treatment approaches include interventional radiologic embolization [12] and surgical ligation of the affected lymphatic channels [13].

Given its established role in the treatment of various benign conditions [14], radiotherapy has also been investigated as an alternative therapeutic option for persistent postoperative lymphatic fistulas [15]. Beyond its anti-inflammatory effects, radiotherapy may promote lymphatic fistula healing through modulation of the vascular endothelium, particularly at low doses [16]. Proposed mechanisms include alterations in endothelial cell adhesion, regulation of cell adhesion molecule expression, and changes in vascular permeability through modulation of intercellular junctions. These effects may enhance lymphatic vessel integrity and reduce lymphatic leakage [17], [18].

Despite these promising biological mechanisms, the clinical use of radiotherapy for postoperative lymphatic fistulas remains limited. The available evidence is sparse, largely retrospective, and often based on outdated and heterogeneous treatment approaches, hindering the development of standardized recommendations and broader adoption in clinical practice.

The present systematic review aims to critically evaluate and synthesize the available evidence regarding the role of radiotherapy in the management of groin lymphatic fistulas, providing a comprehensive overview of all published clinical experiences.

## 2 Materials and methods

This systematic review was conducted in accordance with the Cochrane Handbook of Systematic Reviews of Interventions at each step [19] and adhered to the guidelines outlined in the Preferred Reporting Items for Systematic Reviews and Meta-Analyses (PRISMA) statement [20]. The study protocol was registered within PROSPERO (CRD420261421759).

### 2.1 Inclusion criteria

We included all studies that reported evidence on radiotherapy use for management of postoperative groin lymphatic fistulas. Review articles were excluded.

### 2.2 Information sources and search strategy

A comprehensive search was conducted in MEDLINE via PubMed, from inception until June 2026. The search strategy combined the following keywords and boolean operators: (“lymphatic fistula”[Title/Abstract] OR “lymphorrhea”[Title/Abstract] OR “chylous ascites”[Title/Abstract] OR “lymphocele”[Title/Abstract] OR “lymphatic leakage”[Title/Abstract]) AND (“postoperative”[Title/Abstract] OR “surgery”[Title/Abstract]) AND (“radiotherapy”[Title/Abstract] OR “radiation therapy”[Title/Abstract] OR irradiation[Title/Abstract]).

In addition, a manual search of the reference lists of all eligible articles, relevant review papers, meeting abstracts, and book bibliographies was undertaken to identify further potentially eligible studies.

### 2.3 Selection process

Retrieved records underwent an initial screening based on title and abstract, followed by full-text review for final eligibility assessment. Duplicates within the articles of the literature research were removed. The screening process was verified by a second reviewer, and any discrepancies were resolved through discussion.

### 2.4 Data extraction

Extracted data included study characteristics, patient and fistula numbers, radiotherapy timing and dosimetric parameters, target and irradiated volumes, response assessment, treatment efficacy, time to response, and treatment-related adverse events. The extracted data were checked by the second reviewer, and they were summarized and presented in three tables.

### 2.5 Risk of bias assessment

Risk of bias in the included studies was assessed using a large language model (LLM), verified by one reviewer, applying the JBI Critical Appraisal Checklist for Case Series [21], chosen because all included studies were non-comparative case series or case reports without a control group. The JBI tool provides no quantitative cut-off for risk categorization, leaving this decision to review teams; a methodological analysis of JBI reviews found that most teams define their own cut-off without citing a methodological justification [22]. The review team accordingly adopted, a priori and without validated methodological justification, an 80% threshold (≥8/10 applicable criteria, or the equivalent proportion when items were non-applicable) to define low risk of bias, consistent with other published JBI-based reviews [23], [24], [25], [26]. Studies below this threshold were classified as high risk.

Kapellas et al. [27], a single-patient case report, was instead appraised using the distinct JBI Critical Appraisal Checklist for Case Reports [28]. For descriptive comparison, its appraisal was also mapped onto the Case Series template in Table 4, with inapplicable domains (Q1, Q4, Q5, Q10) marked N/A; this mapped score was excluded from the summary risk-of-bias proportions calculated across the 11 case series.

### 2.6 Statistical analysis

Although meta-analysis was initially considered, it was ultimately deemed inappropriate due to the limited evidence base, which comprised only 12 studies with small sample sizes, substantial heterogeneity in clinical presentation and treatment protocols, and the absence of a standardized definition of treatment success across studies. These limitations also precluded the performance of sensitivity and subgroup analyses, as well as formal assessment of publication bias using methods such as funnel plots or Egger's test. Consequently, the findings are presented as a descriptive synthesis rather than as pooled quantitative estimates.

## 3 Results

### 3.1 Study selection

**Fig. 1:**
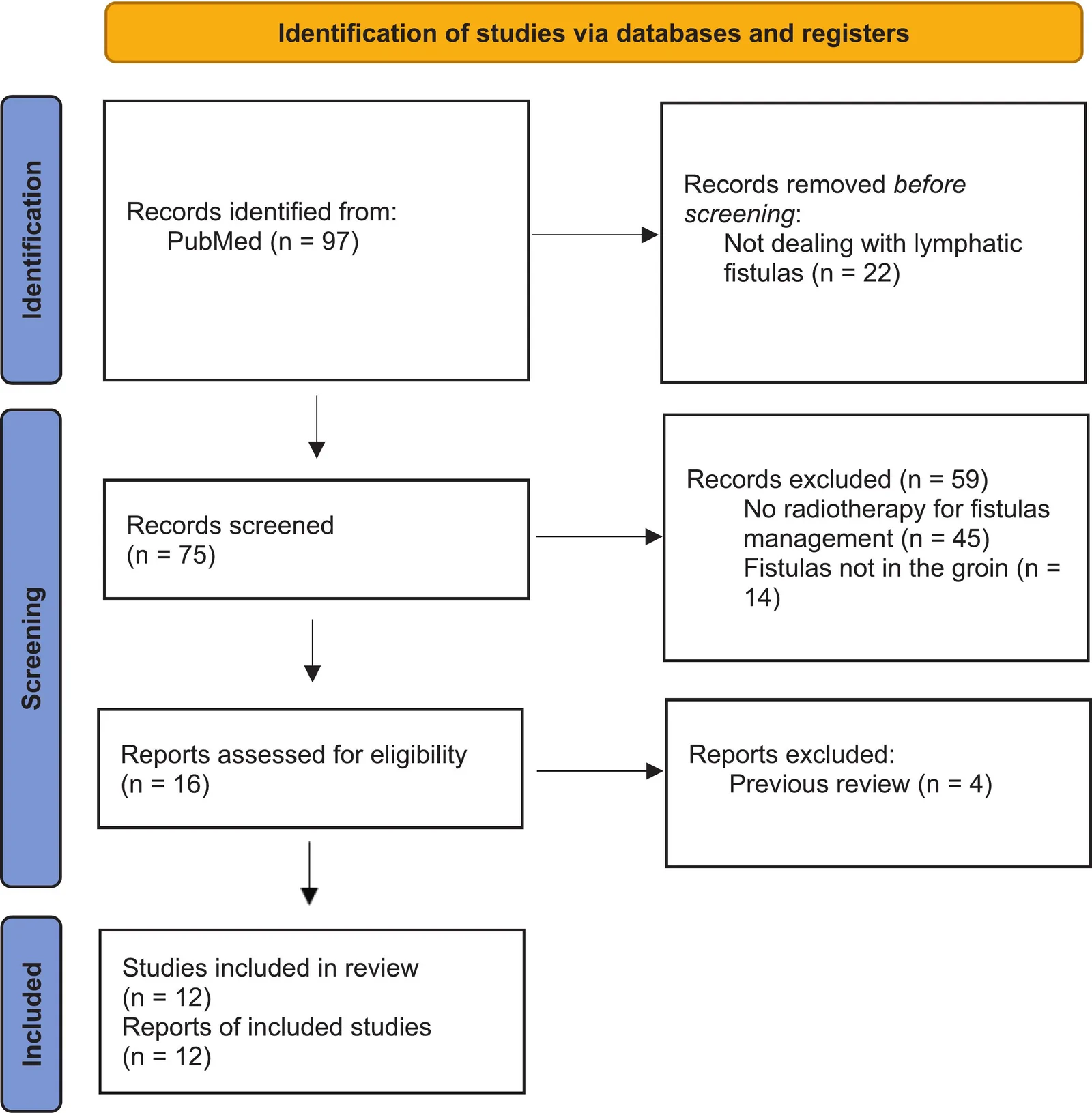
PRISMA flow diagram illustrating the study selection process, including identification, screening, eligibility assessment, and final inclusion in the systematic review.

From 97 identified records, 22 were excluded before screening because they did not directly address the issue of fistula management. Secondly, after screening 75 records, 59 were even excluded because 45 of them did not directly address the issue of radiotherapy for their management and 14 reported experiences for fistulas not in the groin. Finally, 16 records were then assessed for eligibility but 4 previous reviews were excluded because present for three of them [29], [30], [31] an enlarge overview of all lymphatic treatment and for the only specify to radiotherapy a brief report of the few past other studies [1]. The study selection process is shown in Fig. 1.

### 3.2 Study and patient characteristics

Overall, 12 studies published between 1978 and 2026 were included, all of which were retrospective case series and one case report. From all selected studies, 448 patients were included with 1 to 206 fistulas treated per study. In three studies, the number of treated fistulas differed from the number of patients because some individuals underwent bilateral groin irradiation [7], [32], while another study included patients treated for lymphoceles [12]. In the majority of the studies (9 out of 12), the included patients exhibited fistulas that developed following a vascular surgical procedure. Specifically, Röttgen et al. reported only cases of fistula formation subsequent to lymphadenectomy [33], while Kapellas et al. documented a case report involving a patient who presented with a fistula following surgical intervention for inguinal hernia repair [27]. The interval between surgery and radiotherapy was inconsistently reported, ranging from 7.5 to 29 days, with a mean of 17.4 days across studies. Study and patient characteristics are listed in detail in Table 1.

**Table 1:** Treatment time and dose characteristics.

**Study** (first author)	**Treatment period**	**Cause**	**Number of patients** (n)	**Number of fistulas** (n)	**Time to RT** (median in days)	**Beam energy**	**Technique**	**Fraction dose** (Gy/day)	**Total dose planned** (Gy)	**Mean total dose** (Gy) **or fraction delivered** (n)
Croft [15]	1978	Vascular surgery	1	1	27	*NA*	*NA*	3	15	*NA*
Mayer et al. [32]	1987–2002	Vascular surgery Arthroplasty Trauma	17	19	13	Contact therapy (100 Kv) Electrons (4–11 MeV) Photons (8 MV)	2-D	0.3–2	1–12	7 fractions
Neu et al. [12]	1989–1998	Vascular surgery	29	25	*NA*	Electrons (7–18 MeV)	3-D	1	3–12	10 Gy
Dietl et al. [38]	1997–2000	Vascular surgery Trauma	28	28	7.5	Contact therapy (120–300 Kv)	2-D	3	9	3 fractions
Hautmann et al. [7]	2005–2016	Vascular surgery	195	206	10	Photons (6–15 MV)	3-D	3	9	13.8 Gy
Uhl et al. [3]	2007–2018	Vascular surgery	39	39	8	*NA*	*NA*	1	3–10	*NA*
Cañón et al. [37]	2008–2018	*NA*	43	43	25	Photons (6–18 MV)	VMAT	1.5	7.5	*NA*
Buga et al. [34]	2008–2012	Vascular surgery	58	58	*NA*	Contact therapy (180 Kv)	2-D	0.4–0.5	0.8–2	2–5 fractions
Kamalov et al. [35]	2009–2010	Vascular surgery	5	5	*NA*	Contact therapy (180 Kv)	*NA*	0.06–0.3	0.5–4	3–10 fractions
Jazmati et al. [36]	2010–2022	Vascular surgery Node dissection	12	12	*NA*	Photons (6–15 MV)	VMAT	0.3–0.4	3–4	2–10 fractions
Röttgen et al. [33]	2013–2021	Node dissection	20	20	29	*NA*	*NA*	1–1.8	6–20	*NA*
Kapellas et al. [27]	2025	Hernia repair	1	1	20	*NA*	*NA*	0.5	5	10 fractions

*NA*: Not available; n: number, RT: Radiotherapy; Gy: Gray; Kv: Kilovolt; KeV: Kiloelectronvolt; MV: Megavolt; 2-D: 2-Dimensions; 3-D: 3-Dimensions; VMAT: Volume Modulated Arc Therapy.

### 3.3 Radiotherapy characteristics

Regarding the radiotherapy treatment modalities, the selected studies employed various beam energies, including contact therapy, electrons, and photons, from 2-D to 3-D conformal and volumetric modulated arc techniques. Notably, one study utilized three different modalities within the same patient cohort [32]. Among the twelve selected studies, four reported doses in the range of 0.06 to 0.5 Gy per fraction [27], [34], [35], [36], four others used doses between 1 and 2 Gy per fraction [3], [12], [33], [37], and three employed a fixed dose of 3 Gy per fraction [7], [15], [38]. Mayer et al., meanwhile, employed more heterogeneous fractional doses, ranging from 0.3 to 2 Gy per fraction [32]. The total prescribed dose ranged from 0.5 Gy to 20 Gy. Notably, several studies reported discontinuation of radiotherapy once lymphatic drainage ceased, resulting in lower delivered doses than those initially prescribed. Among the included studies, eight explicitly reported the effective total dose or number of fractions truly delivered. Among these, six studies reported a total number of fractions ranging from 2 to 10 [27], [32], [34], [35], [36], [38], while Hautmann et al. documented a mean total dose of 13.8 Gy [7]. Furthermore, Neu et al. noticed a mean total dose of 10 Gy for fifteen patients experienced a significant decrease in lymphatic flow during radiotherapy [12]. Finally, concerning the radiotherapy delivery techniques, it should be noted that the majority of the studies reviewed are relatively dated; notably, only two of these investigations employed Volumetric Modulated Arc Therapy (VMAT) as their technique of choice [36], [37].

Despite substantial missing data and heterogeneity in target volume definitions, the main treatment volume characteristics are summarized in Table 2. Reported target volumes ranged from the surgical scar with a 2–3 cm margin [32], to the fistula and drainage tract with 1 cm margins [38], and to more extensive volumes encompassing the lymphocele, drainage pathway, regional lymphatic structures, or visible tissue alterations with margins ranging from 3 mm to 4 cm [7], [12], [36], [37]. Two studies reported similar median irradiated volumes of approximately 585 cc [7], [36], and again two studies used margins for planning target volume (PTV) between 5 and 10 mm [36], [37]. Radiotherapy characteristics for dose, beam energy and technique are listed in Table 1, and in Table 2 for volumes.

**Table 2:** Treatment volume characteristics.

**Study** (first author)	**Target volume**
Croft [15]	*NA*
Mayer et al. [32]	Scar +2–3 cm margins
Neu et al. [12]	Fistula +4 cm margins
Dietl et al. [38]	Fistula + drainage hole +1 cm margin
Hautmann et al. [7]	Lymphocele + entire tube of the drain + region of the superficial inguinal lymph node + confluence of inguinal veins +1 cm margin
Uhl et al. [3]	*NA*
Cañón et al. [37]	Lymphatic vessels + fistulous tract + associated seroma + scar +3–5 mm margins
Buga et al. [34]	Scar + Fistula
Kamalov et al. [35]	*NA*
Jazmati et al. [36]	Drainage channel + visible tissue alterations +2 cm margin
Röttgen et al. [33]	*NA*
Kapellas et al. [27]	*NA*

*NA*: Not available.

### 3.4 Efficacy and adverse events data

Treatment outcomes are summarized in Table 3, although outcome reporting was incomplete across studies. The most commonly used response criterion was fistula closure, reported in four studies [12], [15], [32], [36]. Other endpoints included drain removal [27], [38], a ≥ 25% reduction in lymphatic secretion after nine days [7], complete cessation of lymphatic discharge [37], complete resolution of lymphatic drainage within 30 days post-irradiation [33], and resolution of lymphorrhea with wound healing after 2–3 sessions [34]. Reported success rates ranged from 67% to 100% and four studies stated a rate of 100%. Among studies reporting response kinetics, the time to complete response ranged from 5.1 to 27 days, with a mean of 11.1 days.

**Table 3:** Treatment outcomes.

**Study** (first author)	**Response criteria**	**Response rate** (%)	**Time to response** (in days after RT start)	**Treatment-related side effects** (%)
Croft [15]	Closure of the lymphatic fistula	100.0	6.0	*NA*
Mayer et al. [32]	Closure of the lymphatic fistula	76.5	13.0	0.0
Neu et al. [12]	Closure of the lymphatic fistula	96.4	NA	*NA*
Dietl et al. [38]	Drain removal	96.4	10.5	0.0
Hautmann et al. [7]	25% reduction of lymph secretion after 9 days	88.3	*NA*	2.4 (cancer development in the pelvic region after RT)
Uhl et al. [3]	*NA*	78.0	*NA*	*NA*
Cañón et al. [37]	Complete cessation of lymphatic discharge	93.0	*NA*	0.0
Buga et al. [34]	Resolution of lymphorrhea and wound healing after 2–3 sessions	67.0	*NA*	0.0
Kamalov et al. [35]	*NA*	100.0	*NA*	*NA*
Jazmati et al. [36]	Closure of the lymphatic fistula	100.0	5.1	0.0
Röttgen et al. [33]	Complete resolution of lymphatic drainage within 30 days post-radiation	84.4	27.0	*NA*
Kapellas et al. [27]	Drain removal	100.0	5.0	*NA*

*NA*: Not available; RT: radiotherapy.

Adverse events reporting was limited. Only Hautmann et al. documented a newly diagnosed pelvic malignancy after radiotherapy, corresponding to a rate of 2.4%, although a causal relationship could not be established [7].

### 3.5 Risk of bias assessment

Risk of bias assessment using the JBI Critical Appraisal Checklist for Case Series is presented in Table 4 for the 11 formally appraised case series.

**Table 4:** Risk of Bias Assessment of Included Studies (JBI Critical Appraisal Checklist for Case Series).

**Study** (first author)	**Q1**	**Q2**	**Q3**	**Q4**	**Q5**	**Q6**	**Q7**	**Q8**	**Q9**	**Q10**	**Overall appraisal (Yes/applicable)**
Croft [15]	N	N	U	N	N	N	U	Y	Y	N/A	2/9 (22%) — High risk
Mayer et al. [32]	U	U	U	U	U	Y	Y	Y	Y	N	4/10 (40%) — High risk
Neu et al. [12]	U	U	U	U	U	Y	Y	Y	Y	N	4/10 (40%) — High risk
Dietl et al. [38]	Y	Y	Y	U	U	Y	Y	Y	Y	Y	8/10 (80%) — Low risk
Hautmann et al. [7]	U	Y	U	Y	Y	Y	Y	Y	Y	Y	8/10 (80%) — Low risk
Uhl et al. [3]	Y	Y	U	Y	Y	Y	Y	Y	Y	Y	9/10 (90%) — Low risk
Cañón et al. [37]	Y	Y	U	U	U	Y	U	Y	Y	Y	6/10 (60%) — High risk
Buga et al. [34]	U	U	U	Y	Y	Y	Y	Y	Y	N	6/10 (60%) — High risk
Kamalov et al. [35]	N	N	U	U	U	N	N	U	Y	N/A	1/9 (11%) — High risk
Jazmati et al. [36]	Y	Y	Y	U	U	Y	Y	Y	Y	N	7/10 (70%) — High risk
Röttgen et al. [33]	Y	Y	Y	Y	Y	Y	Y	Y	Y	Y	10/10 (100%) — Low risk
Kapellas et al. [27] ᵃ	N/A	Y	Y	N/A	N/A	Y	Y	Y	Y	N/A	6/6 (100%) — Low riskᵇ

*Q1 — clear inclusion criteria; Q2 — standard condition measurement; Q3 — valid identification methods; Q4 — consecutive inclusion; Q5 — complete inclusion; Q6 — clear demographic reporting; Q7 — clear clinical information; Q8 — clear outcome/follow-up reporting; Q9 — clear reporting of presenting site(s)/clinic(s); Q10 — appropriate statistical analysis. Y = Yes, N = No, U = Unclear, N/A = Not applicable. Adapted from the JBI Critical Appraisal Checklist for Case Series*[21]*.*

*a — Kapellas* et al. *(n = 1) is a single case report, formally appraised using the distinct JBI Critical Appraisal Checklist for Case Reports*[28]*; it is shown here mapped onto the Case Series template for descriptive comparison only. Q1 (inclusion criteria), Q4 (consecutive inclusion), Q5 (completeness of inclusion), and Q10 (statistical analysis) are marked N/A because they cannot be assessed for a single-patient report by design. b — This 6/6 (100%) score reflects the quality of reporting for this individual case and is excluded from the summary risk-of-bias proportions calculated over the 11 case series (Results).*

Among these 11 series, four (36%) met the pre-defined threshold of ≥80% of applicable criteria and were judged at low risk of bias: Röttgen et al. [33] (10/10, 100%), Uhl et al. [3] (9/10, 90%), and Dietl et al. [38] and Hautmann et al. [7] (8/10, 80% each). The remaining seven (64%) fell below this threshold and were judged at high risk of bias: Jazmati et al. [36] (7/10, 70%), Cañón et al. [37] and Buga et al. [34] (6/10, 60% each), Mayer et al. [32] and Neu et al. [12] (4/10, 40% each), Croft [15] (2/9, 22%), and Kamalov et al. [35] (1/9, 11%).

At the domain level, the use of valid methods for identification of the condition (Q3) was most often unmet or unclear (8 of 11 studies, 73%), followed by consecutive inclusion (Q4) and completeness of inclusion (Q5) (7/11, 64% each), and clarity of the inclusion criteria (Q1) (6/11, 55%). Statistical analysis appropriateness (Q10) was also met in 5 of the 9 studies for which it was applicable (56%).

Kapellas et al. [27], appraised separately as a case report (see Table 4 footnote), met all six applicable mapped domains (6/6, 100%).

## 4 Discussion

Lymphatic fistulas remain frequent complications after vascular surgery or nodal dissection for cancer management [1], [3], [7], [10]. The groin represents the most common anatomical site for persistent lymphatic fistulas, attributable to the high density of lymph nodes and the presence of numerous fine lymphatic channels that facilitate their interconnected network [8]. Lymphatic fistula constitutes the underlying mechanism of three types of lymphatic leakage (lymphatic drainage, lymphocele and lymphocutaneous fistula) and is defined as leakage of lymphatic fluid due to unintended injury of lymphatic channels during surgery [29].

Lymphatic leakage is a well-recognized complication of groin surgery. Following vascular procedures involving a groin incision, leakage within the femoral triangle remains reported in 10–15% of patients despite advances in surgical techniques [10]. Similarly, radical inguinal lymph node dissection for metastatic malignancies of the lower extremities, genital region, or anus is associated with high rates of wound complications, ranging from 14% to 77% across published series [4], [5], [39], [40], [41], [42].

It is noteworthy that there is no universally accepted consensus regarding the precise definition of post-surgical fistulas. Nonetheless, a commonly referenced criterion involves a secretion volume exceeding 50 mL per day, as indicated in several studies [3], [38], although Giovannacci et al. advocate for a lower threshold of 30 mL to characterize such fistulas [43]. The primary point of divergence among definitions pertains to the temporal criterion used to distinguish a persistent postoperative secretion as pathological, thereby identifying it as a fistula. For instance, Dietl et al. consider the fourth postoperative day as the cutoff, Topel et al. regard the seventh day, and Giovannacci et al. define the threshold at the third postoperative day [38], [43], [44]. Gerken et al. propose a nuanced approach by defining a fistula as the persistent secretion of at least 50 mL within 24 h, maintained for more than five days, occurring in the context of lymphatic drainage through surgically inserted drains or from the wound itself in the case of lymphocutaneous fistulas [9], [10]. They proposed also an adapted grading of lymphatic fistulas, with the persistent lymphatic leakage for less than ten postoperative days and the absence of other wound complications for grade A, the persistent lymphatic leakage for more than ten postoperative days or lymphoceles requiring interventions for grade B, and the lymphatic leakage leading to reoperation or subsequent conflict with medical measures or return to normal life for grade C [9].

Lymphatic fistulas may give rise to numerous complications, foremost among which are prolonged hospitalization and an increased frequency of outpatient consultations. The length of in-hospital stays hinges primarily on the presence of wound infections and the progression of lymphorrhea [3]. Although most fistulas tend to resolve spontaneously within a few days, conservative management strategies can be employed. Negative pressure wound therapy (NPWT), for instance, has demonstrated efficacy, with reported control rates ranging from 80% to 100%. However, its application over vascular anastomoses carries a significant risk of hemorrhage [45]. Consequently, surgical revision of the scar is typically reserved for cases complicated by superficial or deep infections of the fistula [46].

Radiotherapy has emerged as a potential alternative treatment, although its radiobiological mechanisms remain incompletely understood. Unlike oncological treatments, radiotherapy for benign conditions is typically delivered at low doses, which are thought to exert anti-inflammatory effects [47]. Low-dose irradiation has been shown to reduce pro-inflammatory cytokine secretion, modulate endothelial permeability, enhance lymphatic reabsorption, and promote tissue fibrosis, all of which may contribute to reducing lymphatic leakage [48]. Despite this biological rationale and several decades of clinical experience, radiotherapy for postoperative lymphatic complications remains supported by limited and heterogeneous evidence and has not been widely adopted in routine practice.

This systematic review synthesized the evidence from 12 studies published between 1978 and 2026. However, the available data remain limited and are derived exclusively from retrospective series, restricting the strength of the conclusions. Despite substantial heterogeneity in treatment techniques, including photon, electron, and contact therapy, the reported outcomes suggest that radiotherapy can be feasibly implemented in most radiation oncology departments.

The present review highlights several important aspects of this treatment modality, particularly regarding dose-fractionation schedules and target volume definition.

A key consideration is the optimal radiation dose prescription. Considerable variability exists in fractionation schedules across studies. Earlier reports generally used fraction doses of 1–3 Gy, in line with conventional radiotherapy practice [3], [7], [12], [15], [37], [38], whereas more recent studies have favored substantially lower doses, typically 0.3–0.5 Gy per fraction [27], [34], [35], [36], and even as low as 0.06 in the study by Kamalov et al. [35]. In an indirect comparison of 17 patients, Mayer et al. observed similar fistula closure rates with low-dose (<0.5 Gy) and higher-dose (>1 Gy) regimens (81% vs. 83%), suggesting that fraction dose escalation may not improve efficacy [32]. Although among the most recent, the study by Röttgen et al. used higher doses, exceeding 1 Gy [33]. However, the oncological context of the patients treated in this study warrants caution when comparing with patients from contemporary studies treated for fistulas occuring mostly after vascular surgery [34], [35], [36].

The optimal total radiation dose remains even less well defined, as treatment in most studies was discontinued once fistula closure was achieved rather than after reaching a predefined dose [35], [36]. Notably, the most recent studies using low-dose fractionation (<0.5 Gy per fraction) reported high rates of complete fistula resolution with cumulative doses not exceeding 4 Gy [34], [35], [36], suggesting that effective outcomes may be achieved with substantially lower total doses than historically prescribed [35], [36]. However, these findings require further validation.

The optimal timing of radiotherapy initiation also remains uncertain. Across studies, median delays ranged from 8 to 29 days, with no clear association between treatment timing and efficacy. While earlier intervention may theoretically be advantageous, available data suggest comparable response rates across different intervals. Uhl et al. who have reported a median delay of 8 days, observed a response rate of 78%, while Cañón et al. found a rate of 93% for a median delay of 25 days [3], [37]. Consistently, Hautmann et al. identified no significant difference in outcomes between patients treated within 5–9 days and those treated later [7], and Neu et al. explained that ten patients enrolled within 14 days of surgery did not experience better outcomes than five patients who had received radiotherapy more than a month after surgery [12]. However, the limited evidence and absence of direct comparisons preclude definitive conclusions.

Last but not least, although the reported CTVs broadly targeted the same anatomical area, notable differences existed in delineation practices, including the consideration of visible tissue alterations [36] and the use of expansion margins ranging from 3 mm to 4 cm. This variability reflects the absence of standardized contouring recommendations for this indication.

Concerns regarding radiation-induced adverse events, particularly secondary malignancies, remain relevant even with low-dose regimens [49]. However, only one case of pelvic cancer following radiotherapy was reported among the included studies [7]. Data on treatment-induced side effects were generally sparse, likely reflecting limited long-term follow-up and under-reporting, which may underestimate late adverse effects including the potential risk of secondary malignancy. Nevertheless, the risk of radiation-induced secondary malignancies appears very low in older patients treated with low-dose radiotherapy for benign conditions [50], especially when peripheral tissues are irradiated.

The risk of bias assessment indicates that only 4 of the 11 case series (36%) met the pre-defined threshold for low risk of bias, indicating that the evidence base for radiotherapy in lymphatic fistula/lymphorrhea remains limited despite the apparent consistency of findings across studies. This predominance of high-risk studies stems largely from two related shortcomings: the diagnostic ascertainment of lymphorrhea was rarely based on an explicit, standardized method (Q3), and case inclusion was often not clearly consecutive or complete (Q4–Q5), leaving open the possibility that favorable outcomes were selectively reported. These design-level weaknesses are compounded by the retrospective, uncontrolled nature of all included studies and by heterogeneous definitions of treatment response across the literature (e.g., fistula closure, drain removal, or a percentage reduction in secretion), which together preclude any pooled or comparative interpretation of efficacy. Two studies illustrate the limits of this evidence base from opposite ends: Röttgen et al. [33], the only case series meeting all ten applicable domains, derived its findings from a distinct clinical population (post-melanoma lymph node dissection rather than vascular surgery), while Kapellas et al. [27], despite being a well-reported single case report, remains by design the lowest level of evidence in the hierarchy; neither, therefore, meaningfully strengthens the overall case-series evidence base.

Finally, this systematic review reveals that the vast majority of patients treated in the included studies underwent irradiation for postoperative fistulas occurring after vascular surgery procedures. In this regard, only Röttgen et al. [33] reported their experience with 20 patients irradiated for fistulas occurring after inguinal lymph node dissection. Although the efficacy rate appears consistent (84%) with other reported outcomes, the applicability of this indication following lymphadenectomy may be questioned, particularly given that adjuvant radiotherapy performed for oncological purposes may have already been administered beforehand.

## 5 Conclusions

This systematic review provides a comprehensive synthesis of the available evidence supporting the use of radiotherapy in the management of postoperative groin lymphatic fistulas. Despite the exclusive availability of retrospective data and the substantial heterogeneity in treatment approaches, reported success rates consistently ranged from 67% to 100%, with a mean time to complete response of approximately 11 days. Low-dose fractionation schedules (0.3–0.5 Gy per fraction) appear to achieve comparable outcomes to higher-dose regimens, with cumulative doses not exceeding 4 Gy demonstrating efficacy in the most recent series. Data on side effects were limited, but the overall safety profile appears favorable.

These findings suggest that radiotherapy represents a feasible, well-tolerated, and potentially underutilized treatment option for refractory postoperative groin lymphatic fistulas, applicable across standard radiation oncology departments without the need for advanced equipment.

However, the lack of prospective evidence, standardized dose-fractionation protocols, and consensus target volume definitions precludes definitive recommendations. Future prospective studies are warranted to establish optimal treatment parameters, including dose prescription, fractionation schedule, target volume delineation, and timing of radiotherapy initiation relative to surgery.

## Use of large language models

A large language model was used to assist in verifying the comprehensive search and the manuscript drafting. It was also used to perform the risk of biais assessment of the included studies using the JBI Critical Appraisal Checklist for Case Series and Case report but with a verification by one reviewver. It does not meet authorship criteria and all content was verified by the authors.

## CRediT authorship contribution statement

**Nicolas Vial:** Conceptualization, Data curation, Formal analysis, Investigation, Methodology, Supervision, Writing – original draft. **Salvatore Cozzi:** Investigation, Validation, Visualization, Writing – review & editing. **Daniele Brenna:** Data curation, Investigation, Validation, Writing – review & editing. **Giulio Brignoli:** Investigation, Validation, Writing – review & editing. **Marialuisa Puglisi:** Investigation, Validation, Writing – review & editing. **Franscesco Mosè Castronovo:** Investigation, Validation, Writing – review & editing. **Davide Giovanni Bosetti:** Investigation, Validation, Writing – review & editing. **Letizia Deantonio:** Investigation, Validation, Writing – review & editing. **Francesco Martucci:** Investigation, Validation, Writing – review & editing. **Calin-Simion Toma-Micle:** Investigation, Validation, Writing – review & editing. **Myriam-Ines Gana:** Investigation, Validation, Writing – review & editing. **Bertrand Le Roy:** Investigation, Validation, Writing – review & editing. **Celine Chauleur:** Investigation, Validation, Writing – review & editing. **Paola Canino:** Investigation, Validation, Writing – review & editing. **Thomas Zilli:** Conceptualization, Investigation, Supervision, Writing – original draft, Writing – review & editing.

## Ethics declaration

The author(s) declare(s) that the study does not involve humans nor animal subjects.

## Funding

No funding.

## Declaration of competing interest

The authors declare that they have no known competing financial interests or personal relationships that could have appeared to influence the work reported in this paper.
